# How to Approach Submucosal Lesions in the Gastrointestinal Tract: Different Ideas between China and USA

**DOI:** 10.1155/2022/8635387

**Published:** 2022-02-24

**Authors:** Rui Ping Gao, Yue Ping Zhang, Qiu Mei Li

**Affiliations:** Department of Gastroenterology, People's Hospital of Ningxia Hui Autonomous Region, 301 Zhengyuan North Street Yinchuan, China

## Abstract

Between 2019 and 2020, the author Gao pursued advanced endoscopic training at the University of Mississippi Medical Center in the USA. She experienced certain different ideas between the East (China) and the West (USA) in terms of endoscopic approach to the submucosal tumors (SMTs) or lesions in the gastrointestinal (GI) tract. In the West (USA), when SMTs are found on gastroscopy, the main goal of endoscopists is to obtain a tissue diagnosis through endoscopic ultrasound-guided fine-needle aspiration or biopsy (EUS-FNA or FNB) or single incision needle-knife biopsy (SINK); if immunohistochemical tests confirmed the GISTs, the first-line treatment is local surgery, that is, diagnosis before treatment, whereas in China, SMTs will be completely resected with endoscopic technology for those with no lymph node metastasis or extremely low risk of lymph node metastasis. There may not be pathological tissue at first, that is, treatment before diagnosis.

## 1. Introduction

Between 2019 and 2020, the author Gao pursued advanced endoscopic training at the University of Mississippi Medical Center in the USA. The author experienced certain different ideas between the East (China) and the West (USA) in terms of endoscopic approach to the submucosal tumors (SMTs) or lesions in the gastrointestinal (GI) tract.

SMT clinically refers to a raised lesion or mass covered by the intact mucosa [1]. In radiology literature, the prevalence of GI SMT is approximately 0.4% [2]. Upper GI SMTs include gastrointestinal stromal tumors (GISTs), leiomyomas, schwannomas, and ectopic pancreas. Most gastric SMTs are benign and can be followed up closely, but GISTs, regardless of their sizes, are currently considered potentially malignant tumors. In clinical practice, SMTs including GISTs are diagnosed by immunohistochemical tests for c-kit, CD34, SMA, S100, etc. Generally, if immunohistochemical tests for c-kit and CD34 are positive, while those for SMA and S100 are negative, the diagnosis of GIST is confirmed. Therefore, immunohistochemistry is particularly important in the diagnosis of gastric submucosal lesions. However, gastric submucosal lesions are located in the submucosal layer and are difficult to obtain by ordinary gastroscopic biopsy. In recent years, the National Comprehensive Cancer Network (NCCN) in the United States and the European Society for Medical Oncology (ESMO) have revised their guidelines for the diagnosis and treatment of GISTs. The GIST diagnosis and treatment guidelines promulgated by these two institutions are the most important practice guidelines for the diagnosis and treatment of GIST in Europe and the United States. The guidelines consider GIST a potentially malignant tumor. Therefore, the first-line treatment of resectable GISTs is local surgery, regardless of the size of the lesion. Therefore, in Europe and America, it is extremely important to obtain pathological tissue from gastric submucosal lesions. At present, the following methods are mainly used to obtain the pathological tissue from gastric submucosal lesions.

## 2. Procedure, Outcome, and Adverse Effect of Endoscopic Ultrasound-Guided Fine-Needle Aspiration or Biopsy (EUS-FNA or FAB)

**Figure 1:**
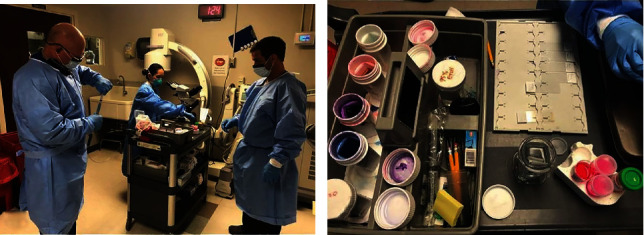
Rapid on-site evaluation.

At present, EUS has been widely used for the diagnosis of pancreaticobiliary diseases. In 1984, Tio and Tytgat described the possibility of using biopsy channels for cytological puncture [3]. In 1991, Caletti et al. were the first to report a case of a patient with gastric SMT who underwent fine-needle aspiration under EUS guidance [4]. Many scholars believe that EUS-FNA/B is a reliable and practical method for evaluating tissues of gastric SMTs [5, 6].When implementing EUS-FNA/B, the patient is placed under general anesthesia, and the procedure needs to be performed under real-time imaging guidance from the linear array ultrasound endoscope. After correctly aiming at the mass, the endoscopist uses the puncture needle to pierce the mass, pulls out the stylet, and connects a 10 ml syringe or uses a microaspiration method with a slow-aspiration needle core. Next, when the assistant uses the connected 10 ml syringe to aspirate, the endoscopist moves the puncture needle back and forth 15-20 times and repeats this operation 2-5 times until enough specimens are obtained. In the endoscopy room of the University of Mississippi Medical Center, rapid on-site evaluation (ROSE) is used to immediately complete the pathological biopsy of the lesion (see Figure 1). If there is no pathologist on-site, the obtained tissue should be immediately placed in the cell block solution for hematoxylin-eosin and immunohistochemical staining to obtain a diagnosis. The 19G Tru-Cut biopsy (TCB) puncture needle is the first device developed for accuracy in obtaining tissue samples [7], but its diagnostic rate for gastric submucosal lesions is only 55%-63% [8, 9]. A recent meta-analysis showed that the puncture needle has an impact on the final pathological diagnosis rate, irrespective of the model (25 G, 22 G, or 19 G) [10]. In order to improve the pathological diagnosis rate, Antonini et al. reported that it is feasible and safe to use a new 20 G puncture needle (Cook Medical, Bloomington, Indiana, USA) in EUS-FNB [11].

EUS-FNA(FNB) is widely used for tissue acquisition of SMTs; however, diagnostic yields for SMTs vary and are relatively low from 74.5% to 83.9% [12–15], particularly for small lesions that are technically challenging to sample using FNA(FNB). A prospective multicencer study by Eckardt et al. revealed a low diagnostic yield of only 52% using a 19 G FNA needle. In the hospital where I studied, I did not see any serious adverse effect.

## 3. Procedure, Outcome, and Adverse Effect of Single Incision Needle-Knife Biopsy (SINK)

**Figure 2:**
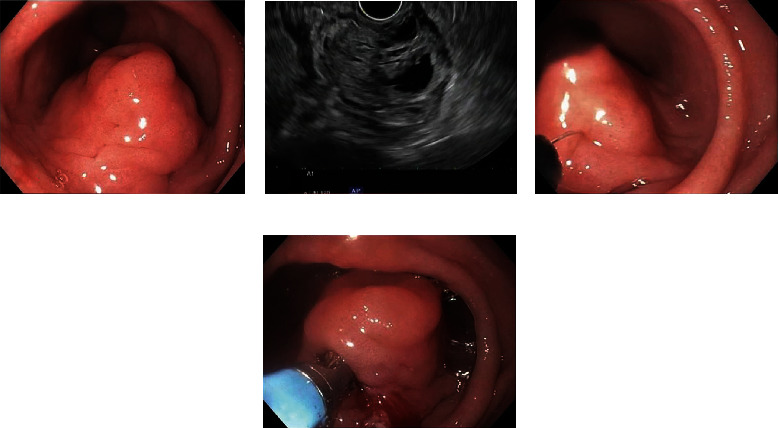
(a) A gastric antral submucosal lesion. (b) Endoscopic ultrasonography shows the origin of the lesions in the muscularis propria. (c) Mucosa incision with needle knife. (d) Biopsy forceps are used for tissue acquisition.

Although EUS-FNA/B is regarded a practical, safe, and effective method to obtain pathological tissues of GI SMTs, this technique involves a ultrasound endoscope, an experienced endoscopist and pathologist, and technical personnel with the ability to use cytology techniques and handle biopsy specimens. Therefore, not every hospital can perform it routinely, and in recent years, single incision needle-knife biopsy (SINK) has been carried out in European and American countries consecutively. In the Endoscopy Room of the University of Mississippi Medical Center, the author observed several cases that used SINK to obtain pathological tissues of gastric SMTs. Once EUS demonstrates that there is no obvious extraluminal compression, lipoma, cyst, or blood vessel, SINK biopsy can be performed. The standard hybrid electrocision was adopted, and then, single incision needle-knife biopsy was performed. A needle knife (Cook Medical) was used to incise the mucosa by about 1 cm and confirmed, under direct vision, to have reached the lesion. Biopsy forceps were then used to remove a few pieces of the lesion. After the operation, an endoclip was used to close the incision to prevent delayed bleeding (see Figure 2), and the patient was observed in the endoscopic room for 1 hour. This method is simple and easy to master. Shimamura et al. reported 49 patients with gastric SMTs who underwent SINK. Histological diagnosis was achieved in all 44 patients. No complications, such as bleeding, perforation, and peritonitis, occurred [16]. De la Serna-Higuera et al. reported 14 patients with SMTs of the upper GI tract. These patients had undergone EUS-FNA, but the pathological specimens were not satisfactory, and the diagnosis could not be confirmed. Therefore, SINK was performed, and sufficiently sized specimens were obtained. Immunohistochemical tests could be performed; 13/14 patients had a clear diagnosis and the diagnosis rate was 92.8%. Therefore, SINK is considered a simple, safe, and effective technique, which can obtain pathological diagnoses of gastric submucosal lesions and help make an assessment on the degree of malignancy [17]. SINK is more likely to acquire adequate tissue for immunohistochemical staining, which may potentially overcome a limitation of EUS-FNA and FNB.

Immediate bleeding at the site of the incision was common, but you can stop it with endoclips. There were no major complications such as perforations or delayed procedure-related complications.

It can be seen from the above methods that in the West, when SMTs are found on gastroscopy, the main goal of endoscopists is to obtain a tissue diagnosis.

To draw comparisons with the West, the author referred to the “Expert Consensus on the Diagnosis and Treatment of Endoscopic Treatment of Submucosal Tumors of the Digestive Tract in China (2018 Edition).” The consensus pointed out that the principle of endoscopic treatment of SMT is “for those with no lymph node metastasis or extremely low risk of lymph node metastasis, that can be completely resected with endoscopic technology, and with a low risk of residue and recurrence, endoscopic resection is suitable. The principle of tumor-free treatment should be followed during endoscopic resection, and the tumor must be completely removed, and the tumor envelope should be intact during resection [18].” The main treatment techniques include endoscopic snare resection, endoscopic submucosal excavation (ESE), submucosal tunneling endoscopic resection (STER), endoscopic full-thickness endoscopic resection (EFTR), and combined endoscopic and laparoscopic technologies. Although these methods may provide complete resection of GIST, there are limitations to these techniques, such as time consuming and having limited application for large tumors (>5 cm) because of a reported perforation rate of up to 19% for larger lesions [19]. Additional risks include positive resection margins, bleeding, and tumor spillage because of a disrupted lesion capsule.

The extensive development of these technologies shows that China's endoscopic diagnostic and treatment technologies have reached a very high level. However, we should also be aware that the diagnosis and treatment levels vary greatly between cities and hospitals of different levels and endoscopists in China. In some large endoscopic diagnostic and treatment centers, submucosal lesions that are found are directly treated by endoscopy after EUS evaluation. As the incidence of complications (bleeding, perforation, peritonitis, etc.) is extremely low, for patients, minimally invasive treatment (ESE, STER, or EFTR) is a great boon. However, most primary hospitals have to consider the feelings of the patients and their families, especially if there is no pathological result before surgery, if serious complications occur during endoscopic treatment, or if there are only small benign lesions after the operation. Therefore, the authors believe that gastric SMT involves gastroenterology and surgery, and extensive communication within multiple disciplinary teams (MDT) should be carried out. From the perspective of the patient's condition, preoperative pathological tests should be completed as much as possible to evaluate the patient's lesion location, size, changes in sonographic images, risk, and metastasis grading of preoperative pathological results, to provide the most suitable treatment.
